# *Acinetobacter baumannii* in the critically ill: complex infections get complicated

**DOI:** 10.3389/fmicb.2023.1196774

**Published:** 2023-06-22

**Authors:** Ilaria Cavallo, Alessandra Oliva, Rebecca Pages, Francesca Sivori, Mauro Truglio, Giorgia Fabrizio, Martina Pasqua, Fulvia Pimpinelli, Enea Gino Di Domenico

**Affiliations:** ^1^Microbiology and Virology, San Gallicano Dermatological Institute, IRCCS, Rome, Italy; ^2^Department of Public Health and Infectious Diseases, Sapienza University of Rome, Rome, Italy; ^3^Department of Biology and Biotechnology "C. Darwin" Sapienza University of Rome, Rome, Italy

**Keywords:** *Acinetobacter baumannii*, cancer, biofilm, skin and soft-tissue infections, colistin, carbapenem, crab, cefiderocol

## Abstract

*Acinetobacter baumannii* is increasingly associated with various epidemics, representing a serious concern due to the broad level of antimicrobial resistance and clinical manifestations. During the last decades, *A. baumannii* has emerged as a major pathogen in vulnerable and critically ill patients. Bacteremia, pneumonia, urinary tract, and skin and soft tissue infections are the most common presentations of *A. baumannii*, with attributable mortality rates approaching 35%. Carbapenems have been considered the first choice to treat *A. baumannii* infections. However, due to the widespread prevalence of carbapenem-resistant *A. baumannii* (CRAB), colistin represents the main therapeutic option, while the role of the new siderophore cephalosporin cefiderocol still needs to be ascertained. Furthermore, high clinical failure rates have been reported for colistin monotherapy when used to treat CRAB infections. Thus, the most effective antibiotic combination remains disputed. In addition to its ability to develop antibiotic resistance, *A. baumannii* is also known to form biofilm on medical devices, including central venous catheters or endotracheal tubes. Thus, the worrisome spread of biofilm-producing strains in multidrug-resistant populations of *A. baumannii* poses a significant treatment challenge. This review provides an updated account of antimicrobial resistance patterns and biofilm-mediated tolerance in *A. baumannii* infections with a special focus on fragile and critically ill patients.

## Introduction

*Acinetobacter baumannii* is an opportunistic pathogen causing severe nosocomial infections (Gonzalez-Villoria and Valverde-Garduno, 2016; Morris et al., 2019). The global estimated incidence rate of *A. baumannii* infections is approximately 1 million cases annually, with high crude mortality rates, particularly in critically ill patients (Peleg et al., 2008; Magill et al., 2014; Lob et al., 2016; Piperaki et al., 2019; Ma and McClean, 2021). Over the last 30 years, *A. baumannii* has emerged as one of the most troublesome pathogens for healthcare institutions, but it rarely causes disease outside of the healthcare setting (Wong et al., 2017). The clinical significance of *A. baumannii* has been raised due to its ability to acquire antibiotic resistance and tolerate desiccation. Indeed, multidrug-resistant (MDR), extensively drug-resistant (XDR), and *A. baumannii* isolates resistant to all clinically available antibiotics (pan-drug resistant—PDR) have been reported worldwide (Piperaki et al., 2019; Weinberg et al., 2020). The rates of MDR are approximately four times higher than those described for other major nosocomial pathogens (Hamidian and Nigro, 2019). Currently, 45% of all *A. baumannii* isolates are classified as MDR, with peaks of 70% in South America, Asia, and Europe (Giammanco et al., 2017; Hamidian and Nigro, 2019). These observations place *A. baumannii* among the most problematic nosocomial ESKAPE (*Enterococcus faecium*, *Staphylococcus aureus*, *Klebsiella pneumoniae*, *Acinetobacter baumannii*, *Pseudomonas aeruginosa*, and *Enterobacter* spp) pathogens, and a “high priority” by the World Health Organization (WHO) and Centers for Disease Control and Prevention [CDC; Tacconelli et al., 2018; Tiku, 2022].

The ability to acquire antibiotic resistance, the environmental persistence, along with the absence of identified toxins in its genome suggest that the virulence potential of *A. baumannii* resides in the ability to survive for prolonged periods throughout a hospital environment (Whiteway et al., 2022). Indeed, adhering to plastics allows *A. baumannii* to colonize endotracheal tubes or central venous catheters, thus increasing its persistence and transmission in hospitalized patients (Peleg et al., 2008; Roca et al., 2012). In particular, *A. baumannii* has been demonstrated to grow as a biofilm on different materials, including health-care-associated equipment, porcelain, stainless steel, rubber, endotracheal tubes, polycarbonate plastic, and polypropylene plastic (Greene et al., 2016a,b). Biofilm formation contributes significantly to establishing medical-device-associated infections conferring a high desiccation resistance and survival of *A. baumannii* isolates (Pour et al., 2011; Greene et al., 2016a,b). Recent reports also suggested that biofilm-producing *A. baumannii* strains are commonly isolated from intensive care units and in oncological patients (Zeighami et al., 2019; Asaad et al., 2021; Di Domenico et al., 2021; Roy et al., 2022). MDR *A. baumannii* (MDRAB) forms robust biofilms, both in the wound and on occlusive dressings in the skin and soft-tissue infections (Thompson et al., 2014). Notably, *A. baumannii* exhibits several adhesive and protective elements that significantly contribute to the formation and maintenance of biofilms, thus increasing tolerance to environmental stressors (Greene et al., 2016a,b). Biofilm is also important to the virulence of *A. baumannii* because it facilitates horizontal gene transfer (HGT) of antibiotic-resistance mobile elements while physically protecting bacteria from the immune system (Eze et al., 2018; Harding et al., 2018).

Infections caused by MDRAB in immunocompromised individuals result from complex relationships between several factors, including *A. baumannii* pathogenicity, the fitness costs of resistance, the site-specific microflora composition of the human host, and the selective forces following clinical interventions such as antibiotic therapy. Therefore, understanding the consequences of mutations driving antibiotic resistance and the worrisome convergence of virulent traits, including biofilm production, has important implications for controlling the spread of *A. baumannii* and developing novel treatment strategies in critically ill patients.

This review provides an updated analysis of antimicrobial resistance mechanisms and biofilm-mediated tolerance in *A. baumannii*. Moreover, we discuss current therapeutic options for carbapenem-resistant *A. baumannii* (CRAB) infections, with a special focus on fragile and critically ill patients.

## Virulence and pathogenicity

Various studies have revealed that *A. baumannii* owns more human virulence potential than other *Acinetobacter* spp. In particular, *A. baumannii* resists macrophage uptake and grows better at 37°C than other species (Tayabali et al., 2012). Some elements, such as the outer membrane proteins (OMP), secretion systems, immunity interaction, or adhesion to the host cells, are highly characterized by virulence and pathogenicity in *A. baumannii* (Morris et al., 2019; Tiku, 2022).

### Outer membrane proteins and outer membrane vesicles

Outer membrane proteins (OMPs) are a class of integral membrane proteins anchored in the outer membrane with a β-barrel structure. OmpA is one of the most abundant porins in the outer membrane of *A. baumannii* (Park et al., 2011; Uppalapati et al., 2020). OmpA is connected to the diaminopimelic acid of the peptidoglycan by two conserved residues (Asp271 and Arg286) in its periplasmic C-terminal domain (Park et al., 2012.). These characteristics give OmpA high stability in the membrane and the capability to fight against harsh environments (Moon et al., 2012). Indeed, being exposed to the outside of the bacterial cell OmpA provides the first line of contact between the bacterium and its surroundings. Given its central position, OmpA acts as an adhesion factor in virulence, channels for the uptake of nutrients, siderophore receptors, and enzymes such as proteases and lipases. Three OMPs were identified as fibronectin-binding proteins, such as OmpA, TonB-dependent copper receptor, and 34 kDa Omp (Smani et al., 2012). OmpA forms a non-selective channel in bacterial outer membranes that permits the passage of ions and other solutes (Sugawara and Nikaido, 2012; Confer and Ayalew, 2013). Furthermore, OmpA contributes to the antimicrobial resistance of *A. baumannii* (Sugawara and Nikaido, 2012; Smani et al., 2014). Indeed, disrupting the OmpA gene decreases the minimal inhibitory concentrations (MICs) of aztreonam, chloramphenicol, and nalidixic acid by 8, 8, and 2.7-fold, respectively. This data suggests that OmpA participates in the extrusion of antibiotics from the periplasmic space through the outer membrane and couples with inner membrane efflux systems (Smani et al., 2014). In *A. baumannii*, OmpA serves multiple functions, both *in vitro* and *in vivo,* including adherence to epithelia, induction of epithelial cell death, drug resistance, channels for the uptake of nutrients, siderophore receptors, binding to factor H (Choi et al., 2005, 2008; Gaddy et al., 2009; Kim et al., 2009). OmpA enhances the survival and persistence of *A. baumannii* by facilitating biofilm formation (Gaddy et al., 2009; Shin et al., 2009). In particular, outer membrane receptor proteins are significantly upregulated in biofilm than in planktonic cultures (Shin et al., 2009). Moreover, it has been reported that overexpression of OmpA represents a significant risk factor for pneumonia, bacteremia, and enhanced mortality in patients infected with *A. baumannii* (Sánchez-Encinales et al., 2017). In *A. baumannii*, virulence factors, including OmpA and certain tissue-degrading enzymes, are delivered to host cells via OMVs (Jin et al., 2011). OMVs are spherical elements with a 20–200 nm diameter, secreted by various Gram-negative pathogenic bacteria (Kulp and Kuehn, 2010). They mainly comprise lipopolysaccharide (LPS), outer membrane and periplasmic proteins, phospholipids, and nucleic acids, representing delivery vehicles for bacterial effectors to host cells (Ellis and Kuehn, 2010). OMVs are central in delivering *A. baumannii* virulence factors, including OmpA, and certain tissue-degrading enzymes, such as proteases and phospholipases (Lee et al., 2017). Furthermore, OmpA has the highest content in OMVs, which is involved in the mitochondrial decomposition of the host’s cell apoptosis (Choi et al., 2005; Tiku et al., 2021).

### Phospholipase

Phospholipases are lipolytic enzyme essential for phospholipid metabolism and a major virulence factor in many Gram-negative bacteria. Phospholipids are the primary building blocks of biological membranes and a carbon and energy source in the human host. In *A. baumannii*, have been identified two phospholipases C (A1S_0043 and A1S_2055) and three phospholipases D (PLD1, PLD2, PLD3), all with substrate specificity toward the eukaryotic membrane component phosphatidylcholine (PC; Flores-Díaz et al., 2016). PC is abundant in eukaryotic membranes representing 50% of all phospholipids and increasing up to 80% in the lung and tracheobronchial secretions (Girod et al., 1992; Bernhard et al., 2001; Tomaras et al., 2003) Experimental evidence suggests that it may serve as a nutrient source during lung infections by pathogens like *Pseudomonas aeruginosa* and *A. baumannii* (McConnell et al., 2013; Sun et al., 2014; Özarslan et al., 2023). Phospholipids’ degradation compromises the stability of host cell membranes, interfering with cellular signaling, thus resulting in changes in the host immune response (Flores-Díaz et al., 2016). In particular, the 1,2-diacylglycerol released by cellular phospholipases C plays roles in modifying biophysical membrane properties, including charge, fluidity, and permeability, and can recruit cytosolic proteins that induce spatial reorganization of signaling complexes, which in turn affect diverse cellular processes (Toker, 2005; Flores-Díaz et al., 2016). Consequently, products generated by bacterial phospholipases could affect the immune response and promote the infection’s establishment or progression (van der Meer-Janssen et al., 2010). In *A. baumannii,* phospholipases D concertedly promote serum resistance, epithelial cell invasion, and *in vivo* pathogenesis (Jacobs et al., 2010; Stahl et al., 2015). Interestingly, PLD1 and PLD2 appear to result from a gene duplication characterized by the HxKx4Dx6GSxN (HKD) pattern similar to eukaryotic cells and required for catalytic activity (Stahl et al., 2015). Despite their similarity, PLD2 is more important for invasion and virulence than the other two PLDs (Jacobs et al., 2010; Stahl et al., 2015). Since phospholipases are conserved across numerous strains of *A. baumannii* and are essential for host invasion, they may represent promising targets for developing enzyme inhibitors and potential vaccine candidates to limit the impacts on human diseases (Flores-Díaz et al., 2016).

### Protein secretion systems

The Type II secretion system (T2SS) is a two-step process, dependent on the general secretory pathway (Sec) or the Twin-arginine (Tat) system for substrate translocation to the periplasm before secretion in the extracellular environment (Weber et al., 2017). The T2SS was first described in *A. baumannii* ATCC17978, with the specific apparatus encoded by genes designated, general secretory pathway (GspA-O), located in six separate operons (Eijkelkamp, 2014). Secretion of type II effector proteins includes enzymes such as lipase, elastase, alkaline phosphatase, and phospholipases, which are essential for *A. baumannii* virulence (Elhosseiny and Attia, 2018). In *A. baumannii*, major T2SS effectors include the metalloendopeptidase, CpaA, and the lipases, LipA and LipH (Johnson et al., 2016; Weber et al., 2017). Secretion of CpaA and LipA requires specific membrane-associated chaperones CpaB and LipB (Zheng et al., 2013; Harding, 2016). In particular, LipA contributes to extracellular lipolytic activity by using long-chain fatty acids as carbon sources for growth and may use fatty acids derived through lipid hydrolysis as signaling molecules allowing bacterial escape from innate immunity (Johnson et al., 2016; Lee et al., 2017). In addition, CpaA is a zinc-dependent metalloendopeptidase forming an active complex with its chaperone (CpaAB), essential for secretion. It targets the common coagulation pathway by interfering with fibrinogen, factor XII and factor V, disrupting blood clotting and allowing the dissemination and colonization of *A. baumannii* (Waack et al., 2018; Urusova et al., 2019). Moreover, mutations in gspD and lipA showed a significant virulence reduction in both *G. mellonella* and murine models (Harding, 2016; Johnson et al., 2016).

Previous studies showed that *A. baumannii* strains produce a type VI secretion system (T6SS) involved in interbacterial competition (Fitzsimons et al., 2018). The T6SS is a complex nanomachine structurally and mechanistically analogous to an intracellular membrane-attached contractile phage tail (Cianfanelli et al., 2016). T6SS is an efficient weapon that can inject toxic effectors into the extracellular environment or directly into eukaryotic or prokaryotic cells (Cianfanelli et al., 2016). In addition, this system is implicated in bacterial competition and DNA uptake released by the prey cells, which promotes horizontal gene transfer (HGT; Weber et al., 2017). Indeed, HGT plays a significant role in the spread of antibiotic resistance cassettes and pathogenicity islands. Therefore, the potential involvement of T6SS in acquiring antibiotic resistance in *A. baumannii* has attracted considerable attention (Weber et al., 2017). It remains to be determined what, if any, benefit the T6SS may provide to *A. baumannii* during infection. In particular, *G. mellonella* infected with *A. baumannii* defective for the T6SS did not succumb to infection as quickly as did worms infected with the wild-type but were killed to the same extent at later time points (Repizo et al., 2015).

## Multiple antibiotic-resistance mechanisms

Increasing reports of the hospital- and community-acquired MDRAB infections are accumulating worldwide (Assimakopoulos et al., 2019; Girija and Priyadharsini, 2019; Darby et al., 2023; Mangioni et al., 2023). In addition to its intrinsic resistance to antibiotics, *A. baumannii* can acquire new functions by HGT, enabling rapid dissemination and maintenance of resistance genes between different isolates (Decré, 2012). Indeed, the European Centre for Disease Prevention and Control’s (ECDC) reported that from 2012 to 2020 in Europe, there had been an increase of 3.4% of *A. baumannii* strains resistant to fluoroquinolones, aminoglycosides, and carbapenems and an alarming rise of 11.3% (217 to 2,451 isolates) in Italy only.

### Fluoroquinolones

**Figure 1:**
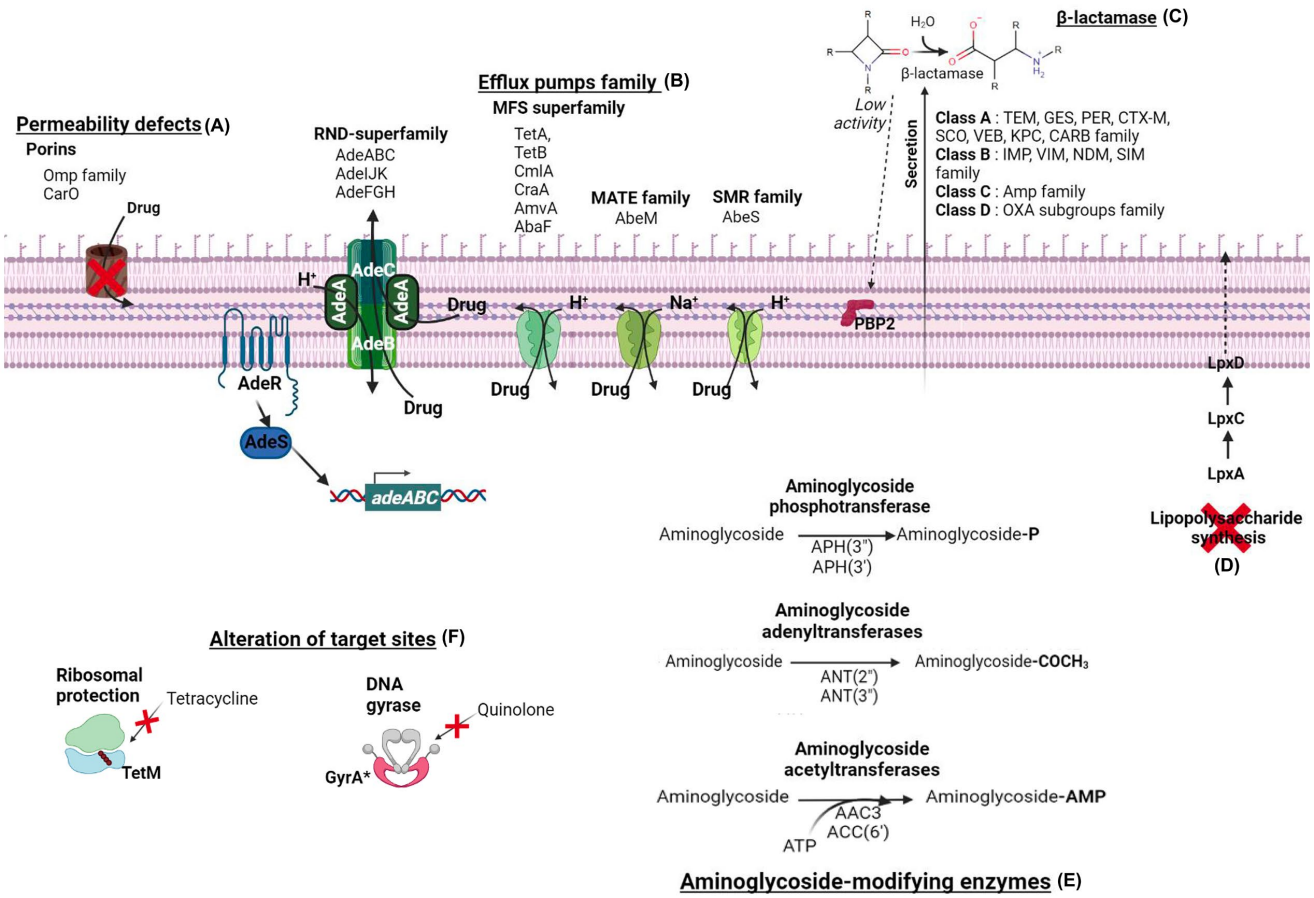
The main antibiotics resistance mechanisms of *Acinetobacter baumannii.* The resistance mechanisms are divided into six categories. **(A)** The permeability defects are due to porins modification, such as the carbapenem-associated outer membrane protein (CarO) and the OMP family. **(B)** The one-step or two-step drug extrusion from the cytosol to the outer membrane via the efflux pumps family. Among them, the resistance-nodulation-division superfamily (RND-superfamily) takes over the drug from the cytoplasm or the periplasm by its AdeABC, AdeIJK, or AdeFGH efflux pumps system. The major facilitator superfamily (MFS; e.g., TetA, TetB, CmlA, CraA, AmvA, AbaF), the multidrug and toxic compound extrusion (MATE) transporter family (e.g., AbeM), and the small multidrug resistance (SMR) transporter (e.g., AbeS) are H+ and Na+ coupled multidrug efflux pumps at the inner membrane. **(C)** The hydrolysis of β-lactam antibiotics by β-lactamases. *Acinetobacter baumannii* β-lactamases are classified into four molecular classes: class A (e.g., TEM, GES, PER, CTX-M, SCO, VEB, KPC, CRAB enzyme family), class B (e.g., IMP, VIM, NDM, SIM enzyme family), class C (e.g., Amp family) and class D (e.g., OXA subgroups enzyme family). **(D)** The complete loss of LPS by inactivating the lipid A biosynthesis genes (*lpxA, lpxC*, and *lpxD*) results in colistin resistance. **(E)** The aminoglycoside-modifying enzymes classified in three class acetyltransferases [e.g., AAC3, AAC(6′)], adenyltransferases [e.g., ANT(2″), ANT(3″)], and phosphotransferases [e.g., APH(3″), APH(3′)]. **(F)** The alteration of targeted sites of TetM confers ribosomal protection against tetracycline, and GyrA subunit modification of DNA gyrase confers resistance to quinolone.

The quinones/fluoroquinolones are antibiotics that inhibit two enzymes involved in DNA synthesis: DNA gyrases and Topoisomerase IV. *A. baumannii* has genetic mutations providing resistance. Mutations in the *gyrA* and *parC* genes of the DNA gyrase subunit and Topoisomerase IV subunit C play a major role in conferring direct antibiotic resistance (Roy et al., 2021). Other important antibiotic resistance mechanisms of *A. baumannii* involve efflux pumps, permeability defects, and alteration of the target site (Figure 1). More generally, three resistance-nodulation cell division (RND)-family efflux pump systems, such as AdeABC, AdeFGH, and AdeIJK, and the multi-antimicrobial extrusion protein family (MATE) efflux pump in *A. baumannii* are overexpressed due to amino acid substitutions in their regulatory genes (Fernandez and Hancock, 2013; Sun et al., 2014; Darby et al., 2023). These two systems allow a broad spectrum of antibiotic resistance to aminoglycoside, chloramphenicol, erythromycin, tetracycline, and tigecycline (Magnet et al., 2001; Fournier et al., 2006; Vila et al., 2007). The plasmid-encoded *qepA* gene is an efflux pump belonging to the major facilitator superfamily that decreases susceptibility to hydrophilic fluoroquinolones, especially ciprofloxacin (Jacoby et al., 2014). With less antibiotic resistance efficiency, mutations in the aminoglycoside transferase AAC(6′)-Ib-Cr by Tryp102Arg and Asp179Typ substitution permit N-acetylation modification of two fluoroquinolones (ciprofloxacin and norfloxacin; Roy et al., 2021; Venkataramana et al., 2022).

### Aminoglycosides

The aminoglycosides antibiotic family inhibits protein synthesis by binding to the 16S ribosomal RNA of the 30S ribosome, with high affinity. Two main mechanisms, involving aminoglycoside modifying enzymes and RNA 16S methylase modification, are associated with increased resistance. Several reports reviewing clinical *A. baumannii* isolates find a match in genes coding for aminoglycosides enzymes modification *ant(3″)-I, aac(3)-I, aph(3′)-I, aac(6′)-Ib* and *aph(3′)-IIb*; and a gene coding for an rRNA 16S methylase *armA* allowing a high antibiotic resistance (Nie et al., 2014; Hasani et al., 2016).

### β-lactam resistance in *Acinetobacter baumannii*

Carbapenems are the most important class of antibiotics against *A. baumannii* and, generally, for Gram-positive and negative isolates (Meletis, 2016). Indeed, carbapenems are considered the drugs of choice to treat *A. baumannii* infections and the first-line agents for empirical therapy in areas with low rates of resistant strains (Pandey and Cascella, 2022). However, different mechanisms of β-lactam resistance have been described resulting in overexpression of OXA β-lactamases and chromosomal cephalosporinases, which have been classified as *Acinetobacter*-derived cephalosporinases (ADCs; Paton et al., 1993). The ADCs overexpression is caused by an insertion sequence (ISAba1) close to these resistance genes (Heritier et al., 2006). The first ADC gene was reported in Spain in 2000 (Bou and Martínez-Beltrán, 2000). Currently, several variants have been described worldwide conferring resistance against penicillins, extended-spectrum cephalosporins, monobactam (aztreonam), and β-lactamase inhibitors (sulbactam; Rodríguez-Martínez et al., 2010; Tian et al., 2011; Kuo et al., 2015; Ingti et al., 2020). The extensive use of carbapenems has been regarded as one of the main risk factors promoting the emergence and spread of MDRAB (Garnacho-Montero et al., 2015). The most effective resistance mechanism is the acquisition of carbapenem-hydrolyzing enzymes. In CRABs, the most common are class D oxacillinases (OXA type) β-lactamases classified in subgroups, with more than 400 OXA-type enzymes identified. Specifically, OXA-23, OXA-24, OXA-51, and OXA-58 subgroups are widespread in *A. baumannii* (Evans et al., 2013). Nevertheless, other β-lactamases classes are involved in carbapenem resistance, such as class A β-lactamases and class B metallo-β-lactamases (MBLs; Smet et al., 2008). OXA-type β-lactamases (especially OXA-23) have also been commonly detected in cefiderocol-resistant *A. baumannii* clinical isolates (Iregui et al., 2020; Kohira et al., 2020; Abdul-Mutakabbir et al., 2021; Yamano et al., 2021). Moreover, PER-like β-lactamases and, to a lesser extent, NDM β-lactamases have been shown to contribute to a decreased susceptibility to cefiderocol (Poirel et al., 2021). Therefore, combined factors, including the presence of β-lactamases such as NDM-like enzymes, modification of the penicillin-binding proteins (target gene PBP-3), permeability defects associated with efflux overexpression and reduced expression or mutation of genes involved in the ion transport, might contribute to resistance to cefiderocol in *A. baumannii* (Malik et al., 2020; Wang et al., 2022). More seldom is the presence of mutations affecting iron transport genes (*pirA* and *piuA*) in cefiderocol-resistant *A. baumannii* isolates (Malik et al., 2020). The *pirA* and *piuA* genes encode components of the pyoverdine and ferric iron uptake systems, respectively. Cefiderocol is transported across the outer cell membrane via iron transporters; thus, mutations in these genes may reduce antibiotic susceptibility. Nevertheless, the finding that mutations in these iron transport genes are relatively rare in *A. baumannii* isolates may suggest that iron acquisition is central to *A. baumannii* survival and, at the same time, genes involved in drug efflux, cell envelope modification, and cell wall biosynthesis may be more efficient in providing resistance to cefiderocol (Moynié et al., 2017).

### The emergence of colistin resistance in multidrug-resistant isolates

**Figure 2:**
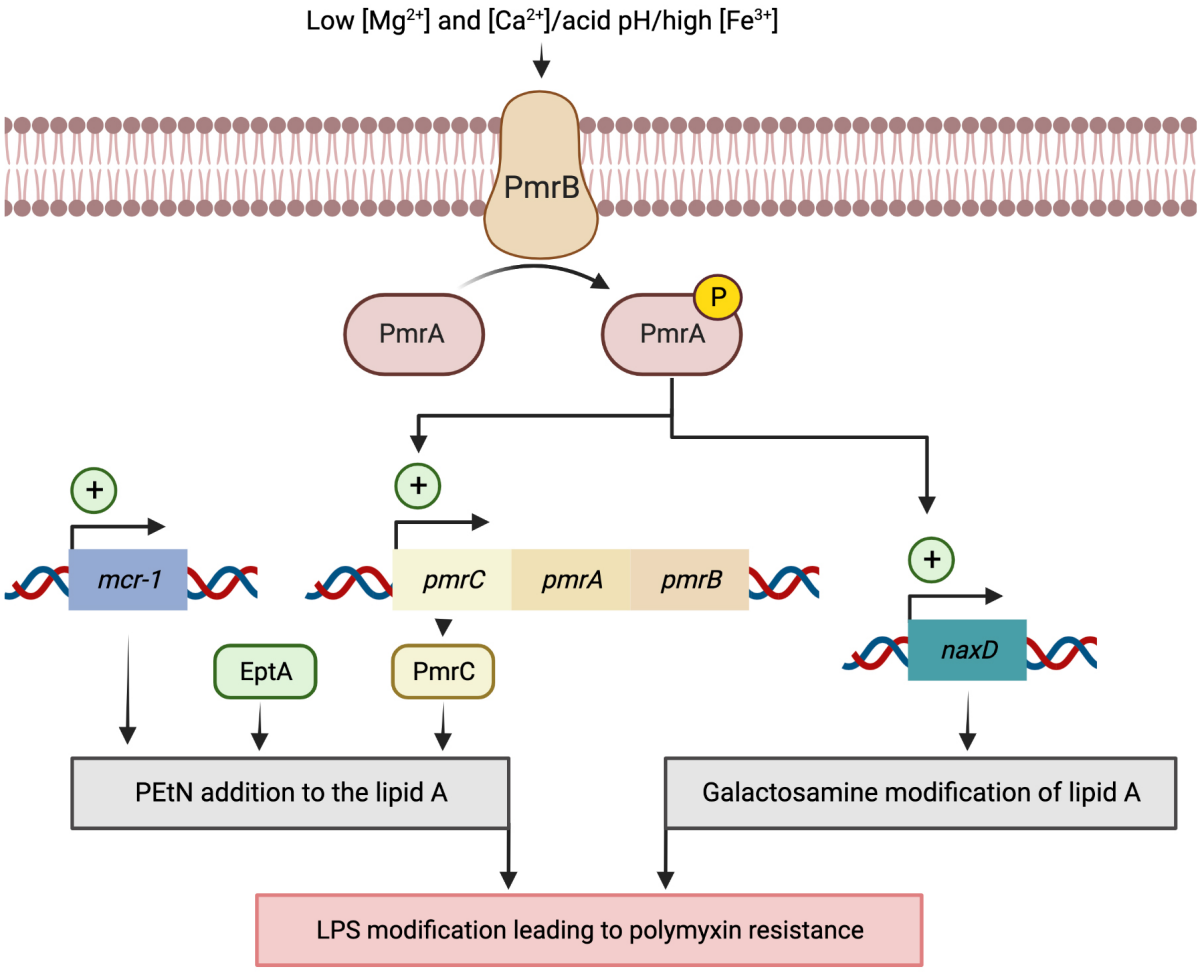
Model for activation of the polymyxin resistance PmrA/PmrB two-component system in *Acinetobacter baumannii*. Resistance to polymyxins can be induced in response to various stress conditions, such as low Mg2+ and Ca2+ concentrations, acidic pH, and high Fe3+ concentrations, which activate the two-component system PmrA/PmrB. Once activated, PmrA/PmrB upregulates *pmrC* gene expression, which encodes lipid A phosphoethanolamine (PEtN) transferase that promotes the addition of PEtN to lipid A. PmrC upregulates *naxD*, which codes for an N-acetylhexosamine deacetylase involved in the deacetylation of the β-galactosamine and Lipid A modification. Alternatively, overexpression of the *eptA* gene, homolog to PmrC, promotes the addition of the cationic pEtN moiety to the lipid A of LPS. Lastly, the plasmid-mediated mobile colistin resistance (*mcr*) genes encode a phosphoethanolamine transferase that adds PEtN to lipid A residues lowering the binding affinity of colistin to its target site.

The increase in colistin treatments after the rise of CRAB has led to a critical emergence of resistant strains, particularly in the hospital environment (Katip et al., 2021a,b). The first recorded case of a colistin-resistant *Acinetobacter* sp. was in 1949 in the Czech Republic (Sun et al., 2020). Currently, the high-resistant clonal lineage of *A. baumannii* has been described across 12 hospitals in Italy, Greece, and Spain, with resistance rates for colistin of 50% (Nowak et al., 2017). Moreover, 42% of *A. baumannii* isolates causing bloodstream infections in intensive care unit (ICU) patients from a Greek hospital have been found resistant to colistin and directly linked to fulminant septic shock and high mortality (Papathanakos et al., 2020). Despite that discovery, the resistance mechanisms to colistin in *A. baumannii* are only partially understood. Colistin is positively charged and interacts electrostatically with the negatively charged phosphate groups of lipid A, the LPS component of Gram-negative bacilli outer membrane. Colistin’s binding causes displacement of calcium (Ca^2+^) and magnesium (Mg^2+^) ions, associated with lipid A phosphoresters, thus affecting the stability of the LPS molecules. Subsequently, colistin inserts its hydrophobic terminal acyl fatty chain, causing disruption and permeabilization of the outer membrane. When permeabilization occurs, colistin penetrates the outer membrane, affecting the integrity of the inner membrane’s phospholipid bilayer, leading to membrane destabilization and cell death (Rhouma et al., 2016; Novović and Jovčić, 2023). Unlike Gram-negative bacteria such as *Salmonella* spp., *Escherichia coli, Klebsiella pneumoniae,* and *Pseudomonas aeruginosa*, *A. baumannii* does not possess a PhoP/PhoQ two-component system. The primary polymyxin resistance mechanisms in *A. baumannii* relies on the PmrA/PmrB two-component system. The PmrA/PmrB is a major regulatory system implied in the lipid A modification (Hua et al., 2020) and is well-characterized in *E. coli*, *P. aeruginosa,* or *K. pneumoniae* (Chen and Groisman, 2013). The histidine-kinase PmrB sensor reacts to various stress conditions, such as low Mg^2+^ and Ca^2+^ concentrations, acid pH, and high Fe^3+^ concentrations (Figure 2).

In Gram-negative bacteria, resistance to polymyxins results mostly from LPS modifications, which is the drug target. These modifications originate from the addition of cationic groups such as 4-amino-L-arabinose (L-Ara4N) and/or phosphoethanolamine (PEtN) on the lipid A (Ezadi et al., 2019). Unlike Enterobacterales, *A. baumannii* lacks all the genes of the *arn* operon required for L-Ara4N biosynthesis. Consequently, colistin resistance is caused by the addition of PEtN to the lipid A on position 1 or 4′ by the chromosomally-encoded EptA-like phosphoethanolamine transferase by the *pmrC* gene (El-Sayed Ahmed et al., 2020).

Mutations in the PmrAB system have been found in a large number of colistin-resistant *A. baumannii* isolates. These mutations constitutively activate the PmrAB regulatory system, which in turn, upregulates the expression of the operon *pmrCAB* (Adams et al., 2009). The self-regulation of the *pmrCAB* transcription enables the modification of lipid A (Olaitan et al., 2014). The colistin resistance-related mutations in the coding sequence for the amino acids Pro102 and Ile13 of PmrA and Pro233, Thr235, and Gln270 of PmrB caused an overactivity of PmrA. These mutations in *pmrA* were located in the sulfatase domain, while in *pmrB* were in the histidine kinase domain. Mutations in *pmrA*-*pmrB* promote phosphorylation of the PmrB receptor kinase, activating PmrA. The activated PmrA modulates the expression of the *pmrC* gene that encodes the phosphoethanolamine transferase that catalyzes the addition of PEtN to the 1′- or 4′-phosphate group of lipid A (Sun et al., 2020). Another study, analyzing the genetic determinants associated with colistin resistance in *A. baumannii* isolates collected from various regions of Greece, identified additional mutations in PmrB (Glu140 or Leu178) and PmrA (Lys172 or Asp10) genes (Palmieri et al., 2020). Besides, PmrA also regulates the *naxD* transcription coding for an N-acetylhexosamine deacetylase which is involved in the deacetylation of the β-galactosamine modifying the Lipid A (Adams et al., 2009; Moffatt et al., 2010; Llewellyn et al., 2012; Deveson Lucas et al., 2018; Sun et al., 2020; Ilsan et al., 2021).

Recently, a plasmid-mediated resistance to polymyxin has been described in Enterobacterales. The *mcr* (mobile colistin resistance) genes also encode a phosphoethanolamine transferase that adds PEtN to lipid A (Partridge et al., 2018). The *mcr-1* remains the predominant plasmid-mediated colistin resistance gene, while *mcr*-2, -3, -4, -5, -6, -7, and -8 have been detected in isolates from animals, humans, and different environments worldwide. Currently, 56 *mcr* variant sequences are available in GenBank (Partridge et al., 2018). The *mcr* genes initially found in Enterobacterales, have been only recently described on *A. baumannii* (Ma et al., 2019; Al-Kadmy et al., 2020), for which resistance to polymyxin was previously restricted to chromosome-encoded elements (Jeannot et al., 2017; Poirel et al., 2017; Partridge et al., 2018).

More recently, a colistin-resistant mutation has also been shown in *A. baumannii* by insertion into the *hns* gene, an H-NS family transcriptional regulator. That mutation alters the expression of more than 150 genes, including the *eptA* gene. Overexpression of this LPS modifying enzyme codes for EptA, a PEtN transferase homolog to PmrC, which confer colistin resistance (Deveson Lucas et al., 2018; Trebosc et al., 2019; Palmieri et al., 2020; Ilsan et al., 2021).

Instead of lipid A modification, *A. baumannii* can acquire resistance to colistin due to the complete loss of LPS by inactivating the lipid A biosynthesis genes (*lpxA*, *lpxC*, and *lpxD;* Moffatt et al., 2010; Cafiso et al., 2019). LpxA, LpxC, and LpxD are three enzymes involved in the first main steps of LPS biosynthesis of *A. baumannii* occurring in the cytoplasm compartment (Powers and Trent, 2018). Specifically, mutations in LpxA and LpxC can lead to modifications in the fatty acid chains of lipid A, while mutations in LpxD can affect the addition of PEtN groups to lipid A. These changes can reduce the outer membrane’s net negative charge and permeability, decreasing colistin susceptibility (Palmieri et al., 2020).

## Bacterial attachment and biofilm formation

*A. baumannii* forms biofilms on a wide range of surfaces, including medical and ventilator-associated pneumonia (VAP), as well as on host epithelial cells leading to meningitis, pneumonia, urinary tract infection, sepsis, and other conditions (Greene et al., 2016a,b; Wong et al., 2017). Biofilm contributes to *A. baumannii* survival on surfaces and in dry and nutrient-deprived conditions for several weeks (Orsinger-Jacobsen et al., 2013; Chapartegui-González et al., 2018). The current understanding suggests biofilm formation in *A. baumannii* is a complex process mediated by a large repertoire of molecules and two-component systems (Mcconnell et al., 2013; Wong et al., 2017; Roy et al., 2022).

### Early surface colonization

Generally, biofilm production relies on the initial reversible bacterial attachment to a surface in response to environmental stimuli (Toyofuku et al., 2016). Thus, early adhesion is essential in the colonization process and in establishing an *A. baumannii* infection. The CsuA/BABCDE chaperon-usher assembly system encodes for the bacterial pili that mediate the attachment of *A. baumannii* to various abiotic surfaces (Longo et al., 2014). The Csu pili comprise four protein subunits, CsuA/B, CsuA, CsuB, and CsuE, assembled via the chaperone–usher pathway (Tomaras et al., 2008). The CsuC chaperone assists the CsuA/B polymerization in forming the major pilus subunit (Pakharukova et al., 2015). In addition, CsuD functions as the usher, CsuE forms a tip adhesin, while CsuA and CsuB constitute minor pilin subunits (Tomaras et al., 2003, 2008; Pakharukova et al., 2015). Previous studies showed that the inactivation of the *csuE* gene abolishes pilus production and biofilm (Amala Reena et al., 2017; Ghasemi et al., 2018). However, a study conducted with 52 different clinical strains revealed that biofilm formation and the ability to attach host cells are independent abilities and not necessarily associated (Eijkelkamp et al., 2011). Indeed, most *A. baumannii* carry the csuA/BABCDE locus; nevertheless, a subset of clinical isolates is *csu* deficient, indicating that these pili may be dispensable for biofilm formation and maintenance and that other pili systems may functionally replace them (Wright et al., 2016). In *A. baumannii,* the expression of the *csu* operon is mainly regulated by the two-component system BfmRS where BfmS acts as a sensor kinase and BfmR functions as a response regulator (Tomaras et al., 2008; Gaddy and Actis, 2009). Indeed, the two-component system BfmRS is considered the master regulator of resistance to stress in *A. baumannii* (Law and Tan, 2022). BfmrR-P can act directly or indirectly on regulating genes for osmotic and oxidative stress, heat shock, the biosynthesis of siderophores, and the production of capsular polysaccharides, in addition to pili production. Furthermore, BfmR is also important for pellicle formation in *A. baumannii* (Krasauskas et al., 2019). A pellicle is an alternative biofilm growing at the air-liquid interface that may favor the colonization and persistence of *A. baumannii* in respiratory tracts, humidifiers, and moist surfaces (Martí et al., 2011; Nait Chabane et al., 2014).

### Biofilm maturation

The two-component system BfmRS is also responsible for the subsequent irreversible adhesion starting with the production of factors under the control and early extracellular DNA (eDNA) release. An early eDNA release was demonstrated to be responsible for the first tridimensional biofilm formation. Notably, eDNA release is independent from the cell lysis in the early stage of biofilm formation and is mediated by membrane vesicles (Sahu et al., 2012).

In *A. baumannii,* the AdeABC, AdeIJK, and AdeFGH RND-type efflux systems are critical in biofilm formation (Coyne et al., 2011). Mutant strains of AdeABC, AdeIJK, and AdeFGH efflux pumps produce a significantly lower level of biofilm than the wild-type strain (Yoon et al., 2015). Moreover, mutation of AdeABC and AdeIJK efflux pumps showed lower expression of several pilus system-encoding proteins, including CsuA/B, CsuC, and FimA. These proteins play a central role in the initial stages of adhesion, surface colonization, and biofilm maturation in *A. baumannii* (He et al., 2015; Shadan et al., 2023).

The AdeRS two-component system regulates the AdeABC efflux pump’s expression (Richmond et al., 2016; Xu et al., 2019). In particular, the deletions of *adeRS* and *adeB* reduced the biofilm growth of *A. baumannii* without affecting the number of adherent cells. This observation suggests that cells might be unable to produce a mature biofilm without this efflux pump (Richmond et al., 2016). After the initial surface attachment, biofilm maturation occurs. During this process, individual cells produce the biofilm matrix entering the irreversible attachment stage. In *A. baumannii,* biofilm maturation is modulated by the Biofilm-associated proteins (Bap) and their interaction with the extracellular polymeric substances (EPS; Soroosh et al., 2020; Upmanyu et al., 2022). The main elements of the *A. baumannii* EPS are alginates and poly-β-(1-6)-N-acetylglucosamine (PNAG) compounds that interact with each other, with ions or heterologous molecules to form an elastic structure (Marvasi et al., 2010). The *pgaABCD* locus is involved in the synthesis of PNAG, facilitating cell adhesion, promoting biofilm integrity, and limiting desiccation (Choi et al., 2009; Morris et al., 2019; Flannery et al., 2020). Accordingly, deleting the pgaABC genes in *A. baumannii* impairs biofilm formation (Choi et al., 2009). The Bap are large surface proteins orthologous to the *Staphylococcus aureus* Bap protein (Cucarella et al., 2001; Loehfelm et al., 2008). A type I secretion system secretes Bap. It is required in cell-to-cell adhesion and for developing higher-order structures on polystyrene and titanium (Loehfelm et al., 2008; Harding et al., 2017). Moreover, the Bap protein increases host colonization by facilitating *A. baumannii* adherence to human neonatal keratinocytes and bronchial epithelial cells (Brossard and Campagnari, 2012). In addition to the Bap protein, the AdeABC efflux pump, normally related to antibiotic resistance, may also contribute to biofilm maturation (Richmond et al., 2016). Notably, in mature *A. baumannii* biofilms, can be observed two types of colonies: the avirulent translucent (AV-T) colonies that produce dense biofilms and virulent opaque (VIR-O) colonies that exhibit low biomass but enhanced virulence in *G. mellonella*, increased surface motility an antibiotic resistance phenotype (Tipton et al., 2015). Several genes are linked to different genomic expression profiles. Among them, ABUW_1132, a highly conserved gene that encodes a LysR-type transcriptional regulator (LTTR) that contributes to the passage of AV-T to VIR-O; its overexpression up-regulates *abaI* and activates the *abaI/abaR* quorum sensing (QS) signal (Tierney et al., 2021). In *A. baumannii*, the QS system is regulated by the two-component system, AbaI/AbaR, which is homologous to the typical LuxI/LuxR system found in other Gram-negative bacteria. *abaI* encodes the autoinducer synthase, which catalyzes the synthesis of N-(3-hydroxy dodecanol)-L-HSL (AHL), which at high density interacts with the cognate receptor AbaR leading to downstream cellular responses (Oh and Han, 2020). Previous studies have found that *abaI* and *abaR* disruption reduces biofilm formation (Niu et al., 2008; Anbazhagan et al., 2012). Moreover, *A. baumannii* cultured in the presence of AHL showed increased expression of Csu pili and biofilm formation (Luo et al., 2015).

### Biofilm dispersion

In the final stage, the cells within the biofilm disperse and colonize new surfaces. Biofilm dispersal is induced prevalently under environmental stress, including the *A. baumannii* SOS response, and the activation of the UmuDAb RecA-dependent repressor inactivated by RecA cleavage when DNA damage occurs. As a result, the UmuDAb mutant cannot activate the transcription of *bmfR.* Thus, no Csu pili or biofilm is formed (Ching et al., 2019). Notably, dispersed cells exhibit variable phenotypes, antibiotic susceptibility, transcriptomic patterns, and metabolic activities (Rumbaugh and Sauer, 2020). For example, dispersed clinical isolates of *A. baumannii* are more hydrophobic and adhere more efficiently to the surface than the planktonic cells (Berlanga et al., 2017). Moreover, the dispersed cells were more susceptible to ciprofloxacin and tetracycline than the same cells in the planktonic state (Berlanga et al., 2017). In contrast, another *A. baumannii* clinical strain disseminating from ciprofloxacin-exposed biofilms is highly resistant to ciprofloxacin, erythromycin, and tetracycline (Penesyan et al., 2019). These studies suggest that the ability of the dispersed cells to evolve, acquiring higher antibiotic resistance, could complicate the management and treatment of the infection (Law and Tan, 2022).

### What is the clinical relevance of biofilm production among patients with *Acinetobacter baumannii* infection?

The ability of *A. baumannii* to form biofilms has been reported as an essential factor contributing to its persistence and tolerance to antimicrobial agents (Roy et al., 2022). The proportion of *A. baumannii* clinical isolates that produce biofilms can vary significantly depending on the study and sample population. In a collection of 20 clinical isolates of *A. baumannii*, emerged that 80% of the strains formed biofilm, perhaps because of a dominant clone (Sechi et al., 2004). Bardbari et al. compared biofilm-production ability between clinical and environmental *A. baumannii*. In this study emerged that the majority of both clinical and environmental isolates could form varying degrees of biofilm. Specifically, the prevalence of strong biofilm producers in clinical and environmental strains was 58.7 and 31.2%, respectively (Bardbari et al., 2017). Others reported that among 154 *A. baumannii* isolated in Taiwan, 45.4% possessed strong biofilm formation ability (Yang et al., 2019). Moreover, among 100 *A. baumannii* clinical isolates from three hospitals in Iran, 58% were strong biofilm producers (Zeighami et al., 2019). Another study investigating 92 unrelated strains of *A. baumannii* isolated from two Spanish hospitals found that 63% of isolates formed biofilm, mainly from device-associated infections. Notably, these isolates were less frequently resistant to imipenem or ciprofloxacin than non-biofilm-forming isolates (Rodríguez-Bano et al., 2008).

A study from 4 Chinese hospitals analyzed the contribution of biofilm formation in the epidemic spread of *A. baumannii* by comparing biofilm-forming abilities and genetic characteristics of international clonal lineage II (ICL II) and non-ICL II isolates. From a total of 114 clinical *A. baumannii* isolates, collected from various specimens, including blood, sputum, urine, and wound, emerged that 36% of the clinical isolates were able to form biofilm, but only 19.5% were strong biofilm producers. Of the *A. baumannii* isolates, the biofilm formation capacity of ICL II was significantly lower than that of non-ICL II isolates. The authors concluded that biofilm formation might not be a critical factor for the epidemic spread of *A. baumannii*, particularly for the ICL II lineage. They suggested that other factors, such as antimicrobial resistance and virulence, could play a more critical role in the epidemic potential of *A. baumannii* (Hu et al., 2016). Despite the propensity to produce biofilm, the clinical impact of biofilm in *A. baumannii* isolates is still debated. Indeed, a recent multicenter study in Taiwan including 711 patients showed that higher APACHE II score, shock status, lack of appropriate antimicrobial therapy, and carbapenem resistance were independent risk factors of 28-day mortality in the patients with *A. baumannii* bacteremia but not the level of biofilm formation. In addition, biofilm formation was most commonly observed in survivors than in non-survivors (38.4% vs. 31.9%; Chiang et al., 2022). Similar results have been previously observed in a cohort of 273 patients with *A. baumannii* bacteremic pneumonia (Wang et al., 2018). Accordingly, other studies have shown that infections caused by biofilm-producing *A. baumannii* are not necessarily associated with worse clinical outcomes (Rodríguez-Bano et al., 2008; Wang et al., 2018). Therefore, the impact and pathogenesis of biofilm production remain elusive and, in many cases, related to the patient’s underlying condition or to the strain that causes the infection (Rodríguez-Bano et al., 2008; Barsoumian et al., 2015; Wang et al., 2018; Di Domenico et al., 2020, 2021).

*A. baumannii* is known for its ability to develop resistance to multiple antibiotics, making treatment of infections particularly challenging. In addition, the formation of biofilms further exacerbates this issue, as the extracellular matrix can act as a physical barrier, limiting the penetration of antibiotics and protecting the bacteria from the host’s immune system (Perez et al., 2007; Antunes et al., 2011). Several antibiotics and antibiotic combinations have shown promise in combating *A. baumannii* biofilms. However, their effectiveness may vary depending on the strain and resistance profile. The use of two or more antibiotics with different mechanisms of action can enhance the therapeutic efficacy by affecting multiple bacterial targets. In particular, the combination of colistin and rifampicin was more effective at eradicating biofilms formed by multidrug-resistant *A. baumannii* isolates than either antibiotic alone (Batoni et al., 2016). The antimicrobial combinations of colistin-levofloxacin, colistin-tigecycline, and tigecycline-levofloxacin or these combinations with clarithromycin were effective as lock solutions in the treatment of *A. baumannii* catheter-related infections (Ozbek and Mataraci, 2013). Nevertheless, candidate antibiotics were active against biofilm-embedded *A. baumannii* cells at 400-fold the MIC. This concentration is unachievable in human serum, making those antimicrobials an undesirable option for systemic use in *A. baumannii* biofilm-associated infections (Ozbek and Mataraci, 2013). Synergistic effects were also observed on biofilm-embedded carbapenem-resistant and carbapenem-susceptible *A. baumannii* strains. In particular, meropenem was active against biofilm-embedded carbapenem-susceptible *A. baumannii*, whereas meropenem plus sulbactam exhibited synergism against biofilm CRAB and caused significantly more damage to the biofilm architecture than colistin or tigecycline used alone (Wang et al., 2016). Additionally, clinical isolates of MDRAB exhibited different degrees of biofilm formation in the presence of sub-minimum inhibitory concentrations of colistin and tigecycline (Sato et al., 2018). A recent study showed that biofilm-embedded MDRAB had been eradicated with colistin but not tigecycline. Notably, the eradication increased with a combination of colistin and high concentrations of tigecycline (Sato et al., 2021). Moreover, combining azithromycin and polymyxin B displayed synergistic activity against biofilm-producing *A. baumannii* clinical isolates, improving antimicrobial efficacy (Peng et al., 2020). These data suggest that the effects of different antibiotics may depend on bacterial strains and the response of *A. baumannii* may vary under specific environmental stress conditions, such as in the presence of multiple antimicrobial agents. Nevertheless, one of the main challenges in analyzing these studies is the considerable heterogeneity in the design, methodologies, and patient populations examined (Table 1). While reflecting the field’s richness, such diversity can make it difficult to draw firm conclusions or compare findings directly across studies. Additionally, there is not yet a universally accepted definition or a standardized method for determining biofilm formation by *A. baumannii*. The absence of such standards introduces variability between studies and complicates the comparison of results. Furthermore, many of our findings are based on *in vitro* studies. While these studies provide valuable insights, they cannot fully capture the complexity of clinical infections. The behavior of *A. baumannii* in a real-world clinical setting can be influenced by myriad factors not present under laboratory conditions.

**Table 1:** Activity of different antibiotics against carbapenem-resistant *Acinetobacter baumannii* (CRAB).

Drug	Mechanism of action	MIC breakpoint for CRAB (EUCAST)§	Side effects	Anti-biofilm activity vs. CRAB	Dosage for CRAB infections
Colistin	Colistin binds to LPS and phospholipids in the outer cell membrane of Gram-negative bacteria	2 μg/mL	Nephrotoxicity, neurotoxicity	No	As per international consensus guidelines (Tsuji et al., 2019)
	It competitively displaces divalent cations (Ca^2+^ and Mg^2+^) from the phosphate groups of membrane lipids, which leads to disruption of the outer cell membrane, leakage of intracellular contents, and bacterial death			Absence of anti-biofilm activity also when combined with meropenem, ampicillin/sulbactam, and minocycline	
				Colistin plus rifampin retains anti-biofilm activity (Wang et al., 2016; Wences et al., 2022)	
Tigecycline	Tigecycline binds to the 30S ribosomal subunit and blocks the entry of amino-acyl tRNA molecules into the A site of the ribosome, inhibiting protein translation in bacteria.	IE	Nausea, vomiting, diarrhea, hepatotoxicity, pancreatitis	No (Wang et al., 2016)	200 mg loading dose followed by 100 mg every 12 h
Ampicillin/sulbactam	Sulbactam is an irreversible competitive beta-lactamase inhibitor that can saturate Penicillin Binding Proteins (PBP) 1 and 3 in *Acinetobacter* spp. when given in high doses	IE	Hepatotoxicity	No	3–9 g every 8 h (for ampicillin-sulbactam 2:1)
	Anti-CRAB activity is exerted by sulbactam.			Meropenem plus sulbactam was synergistic against biofilm-embedded CRAB (Wang et al., 2016; Chaiben et al., 2022)	A high dosage (9 g every 8 h) is required for VAP (Jaruratanasirikul et al., 2019)
Cefiderocol	Cefiderocol is a siderophore cephalosporin actively transported into the periplasmic space of Gram-negative bacteria through the bacterial siderophore iron uptake system, as well as through passive diffusion via outer membrane porin channels	Zone diameters of ≥17 mm for the cefiderocol 30 μg disk correspond to MIC values below the PK-PD breakpoint of S ≤ 2 μg/mL	Elevated liver tests, hypokalemia	Yes (Pybus et al., 2021)	2 g every 8 h infused over 3 h
					2 g every 6 h infused over 3 h if CrCl≥120 mL/min
Fosfomycin	Fosfomycin interferes with the first cytoplasmic step of bacterial cell wall biosynthesis, the formation of the peptidoglycan precursor UDP N-acetylmuramic acid (UDP-MurNAc).	No breakpoint available	Hypernatremia, hypokalemia	Alone: no	12–24 g/die (divided every 8–12 h)
				In combination with colistin: yes (Boncompagni et al., 2022)	
Eravacycline	Eravacycline binds reversibly to the 30S ribosomal subunit, inhibiting protein translation in bacteria.	IE	Gastrointestinal side effects	No data	1 mg/kg/dose every 12 h
Sulbactam/durlobactam	Durlobactam is a novel non-ß-lactam diazabicyclooctane ß-lactamase inhibitor with broad-spectrum activity against class A, C, and D ß-lactamases.	No data	Gastrointestinal side effects	No data	1/1 g every 6 h, according to the ATTACK study (ClinicalTrials.gov: NCT03894046)

CRAB, carbapenem-resistant *A. baumannii*; §, accessed on 7th March 2023; IE, insufficient evidence that the organism or group is a good target for therapy with the agent; CrCl, creatinine clearance.

## *Acinetobacter baumannii* infections and treatment options in critically ill patients

The two most common clinical manifestations of *A. baumannii* are nosocomial pneumonia, particularly VAP, and bacteremia (Wong et al., 2017). While an endotracheal tube allows *Acinetobacter* spp. to establish biofilm facilitating its transmission and spread in the environment, the development of VAP occurs due to the aspiration of bacterial *droplets* directly into the alveoli. Likewise, bacteremia occurs as a hematogenous spread from pneumonia or in the presence of an infected central venous catheter. Less commonly, *A. baumannii* causes urinary tract infections (often associated with the presence of urinary catheters), central nervous system infections (often after neurosurgery or in the presence of external ventricular drain), wound or bone infections (often after surgery or trauma; Wong et al., 2017). Typically, infections sustained by *A. baumannii* occur in intensive care units, where patients are characterized by critical illness, multimorbidity, prolonged hospital stay, exposure to multiple invasive procedures, and prolonged antibiotic therapy (Ogutlu et al., 2014; Ayobami et al., 2020; Ibrahim et al., 2021).

During the COVID-19 pandemic, MDR organisms, particularly CRAB, have been increasingly reported as causative agents of secondary infections, especially in severe and critical diseases (Patel et al., 2021; Cogliati Dezza et al., 2022; Russo et al., 2022; Langford et al., 2023). Furthermore, CRAB acquisition increased during the hospital stay and accounted for high mortality rates in patients with COVID-19 (Falcone et al., 2021; Iacovelli et al., 2023). Increased antibiotic resistance, reported for clinical isolate, is even more significant in oncological patients (Ñamendys-Silva et al., 2015; Nazer et al., 2015; Cornejo-Juárez et al., 2020). A previous report highlights that among 635 oncological patients, 6.1% were infected by *A. baumannii* MDR (Nazer et al., 2015). An oncology department in China demonstrated that *A. baumannii* accounted for 9.8% of infections (Li and Wang, 2018). Two studies have shown that 19% of patients died within 72 h after *A. baumannii* isolation (Nazer et al., 2015; Cornejo-Juárez et al., 2020).

Therefore, CRAB represents a threat to the most vulnerable patients, contributing to the observed high mortality, which reaches values up to 50%–70% in patients with septic shock and VAP (Iovleva et al., 2022). Furthermore, despite sharing similar comorbidities and risk factors, patients infected with CRAB or XDR strains had a significantly higher mortality rate than those caused by susceptible strains (Lee et al., 2014; Lemos et al., 2014). A recent study further highlighted that the absolute excess 30-day mortality due to infection sustained by PDR *A. baumannii* compared to only PDR *A. baumannii* colonization was 34%, suggesting that one of every three treated patients would have been saved if effective drugs were available (Karakonstantis et al., 2020).

Despite being a strong biofilm producer, it has been shown that biomass production was not an independent risk factor for 28-day mortality in patients with *A. baumannii* bacteremia (Chiang et al., 2022). Indeed, one of the major drivers of mortality is the inappropriate initial effective therapy, which mainly depends on the high resistance level in *A. baumannii*. Currently, there is still no consensus on the optimal treatment of CRAB infections (Paul et al., 2022; Tiseo et al., 2022). Colistin has been considered the backbone of CRAB treatment for many years, mostly in combination with carbapenems, fosfomycin, tigecycline, or ampicillin/sulbactam or even with vancomycin and/or rifampin (Durante-Mangoni et al., 2013; Ceccarelli et al., 2015; Oliva et al., 2017; Giacobbe et al., 2020; Katip et al., 2020). Colistin is administered as an inactive prodrug, colistimethate (also known as colistin methanesulfonate, CMS). International consensus guidelines and recent studies highly recommend administering CMS as a loading dose (LD) followed by a maintenance dose for the treatment of infections due to carbapenem-resistant Gram-negative bacilli, especially in critically ill patients (Tsuji et al., 2019; Wang et al., 2022). A recent study evaluated the efficacy and safety of using a CMS LD in the treatment of critically ill patients with CRAB infections and showed higher clinical, microbiological, and 30-day survival rates in patients receiving LD compared with patients not receiving LD; however, the administration of the LD was associated with a higher risk of nephrotoxicity (Katip et al., 2021a,b).

Colistin use is limited by the risk of nephrotoxicity if administered at clinically effective dosage (Ordooei Javan et al., 2015) and the relatively poor lung epithelial lining fluid (ELF) penetration in critically ill patients (Imberti et al., 2010). Furthermore, resistance to colistin may occur in up to 30% of CRAB strains (Iovleva et al., 2022), rendering the treatment of CRAB infections even more challenging. In any case, the rate of colistin resistance is lower than that of tigecycline (45.5%; Chang et al., 2012; Muthusamy et al., 2016), suggesting this antibiotic still represents an effective antimicrobial agent against CRAB infections (Katip et al., 2021a,b).

Sulbactam is an irreversible competitive beta-lactamase inhibitor with direct antimicrobial activity thanks to its intrinsic affinity for the *A. baumannii* PBPs (Tamma et al., 2022). In addition, when given in high doses, sulbactam has the ability to saturate PBP-1 and PBP-3 and may therefore overcome the increasing described rates of sulbactam resistance in CRAB (Bartal et al., 2022).

In recent years, cefiderocol, a novel siderophore cephalosporin, has been approved by the Food and Drug Administration to treat serious infections caused by carbapenem-resistant Gram-negative bacteria (US Food and Drug Administration, 2019) and represented an encouraging advancement, especially for the treatment of CRAB infections. While the phase 3 randomized clinical trial CREDIBLE-CR, which compared cefiderocol with the best available therapy, showed higher mortality in the subgroup of patients with CRAB treated with cefiderocol (Bassetti et al., 2021), subsequent real-world observations from case series or observational studies showed promising results of cefiderocol in terms of efficacy (Oliva et al., 2020; Bavaro et al., 2021; Rando et al., 2021; Falcone et al., 2022) and safety (Pascale et al., 2021). This advantage was more evident in patients with bloodstream infections than those with VAP (Falcone et al., 2022), probably due to a sub-optimal penetration of cefiderocol in the ELF at current dosages (Gatti et al., 2021). However, the possibility of developing resistance to this drug under treatment, associated with an observed higher microbiological failure than the best available therapy (Falcone et al., 2022), requires caution and deserves further prospective studies to define cefiderocol optimal place in therapy toward CRAB infections (Volpicelli et al., 2021).

Given the limited therapeutic options with conventional antibiotics, there is ongoing research on alternative or adjuvant strategies for treating CRAB infections. In particular, N-acetylcysteine (NAC) exhibited high *in-vitro* activity against both planktonic and biofilm CRAB (Pollini et al., 2018; De Angelis et al., 2022), while a recent clinical observation showed a survival benefit of intravenous NAC addition to antibiotics in critically ill patients with CRAB septic shock (Oliva et al., 2021).

## Conclusion

*A. baumannii* has emerged as an opportunistic pathogen responsible for a broad range of severe nosocomial infections. Much of *A. baumannii*’s success can be directly attributed to its genome plasticity, which rapidly mutates under stress. The ability to resist most last-line antimicrobial agents poses a considerable challenge, especially in critically ill patients.

In particular, the dissemination of CRAB and the increase in the use of colistin has led to a critical emergence of resistant strains. However, several virulence mechanisms beyond canonical drug resistance were recently identified, enabling *A. baumannii* to thrive in the healthcare environment. Indeed, it has been observed that *A. baumannii* can contaminate hospital surfaces or devices, caregivers’ hands, and can be spread by asymptomatically colonized persons. In addition, desiccation resistance, surface adherence, and biofilm formation make *A. baumannii* outbreaks in acute care hospitals difficult to control.

The environmental persistence has probably contributed to the increase in the incidence of *A. baumannii* from COVID-19 patients highlighting the value of appropriate prevention and control practices, particularly in open-space ICUs. During the COVID-19 pandemic, decreased vigilance for MDR control of transmissions, suspension or limitation of the hospital infection control committees, reduced surveillance, and personnel numbers likely contributed to the increase in hospital-acquired infections caused by *A. baumannii.* Notably, this review focuses on critically ill patients, a population particularly vulnerable to *A. baumannii* infections. Nevertheless, these infections also occur in other patient populations, and some of the data and conclusions herein presented may not be universally applicable.

Therefore, rapid diagnostic tests to identify and track high-risk clones, and antibiotic resistance genes, together with appropriate antibiotic regimens and strict adherence to infection control measures, may represent priorities for effectively dealing with *A. baumannii* infections.

## Author contributions

IC, AO, FS, GF, MT, MP, and ED contributed to the review’s conception and design. IC, AO, FS, RP, FP, and ED researched and wrote the review. All authors contributed to the article and approved the submitted version.

## Funding

This research was funded by the Italian Ministry of Health (RC 2023) and also supported by EU funding to AO within the NextGeneration EU-MUR PNRR Extended Partnership initiative on Emerging Infectious Diseases (project no. PE00000007, INF-ACT).

## Conflict of interest

The authors declare that the research was conducted in the absence of any commercial or financial relationships that could be construed as a potential conflict of interest.

## Publisher’s note

All claims expressed in this article are solely those of the authors and do not necessarily represent those of their affiliated organizations, or those of the publisher, the editors and the reviewers. Any product that may be evaluated in this article, or claim that may be made by its manufacturer, is not guaranteed or endorsed by the publisher.
